# Clinical data and Pediatric Quality of Life Inventory (PedsQL™) scores for children with duodenal atresia

**DOI:** 10.1016/j.dib.2020.105184

**Published:** 2020-01-25

**Authors:** Toby I. Vinycomb, Alison Browning, Matthew L.M. Jones, John M. Hutson, Sebastian K. King, Warwick J. Teague

**Affiliations:** a Department of Paediatric Surgery, The Royal Children's Hospital, Melbourne, Australia; b Surgical Research Group, Murdoch Children's Research Institute, Melbourne, Australia; c Department of Surgery, University of Sydney, Sydney, Australia; d Department of Urology, University of Melbourne, Melbourne, Australia; e Department of Paediatrics, University of Melbourne, Melbourne, Australia

**Keywords:** Duodenal atresia, Duodenal obstruction, Quality of life, Long term outcomes, Intestinal atresia, Congenital abnormalities

## Abstract

This article presents raw data obtained from a prospectively collected database of children with duodenal atresia at tertiary pediatric surgery hospital. For all potential participants, pertinent demographic, clinical and operative data was obtained from the database. Potential participants were then contacted and invited to complete a Pediatric Quality of Life Inventory (PedsQL™) 4.0 core score and gastrointestinal module questionnaires. Participant's response to each item in the questionnaires is provided, as well as their calculated health related quality of life scores. Data has the potential to be reused in future studies examining quality of life in duodenal atresia, paediatric gastrointestinal conditions, surgical neonatal conditions and children with trisomy 21. Further analysis and discussion is contained in related research article titled “Quality of life outcomes in children born with duodenal atresia” [1].

**Table:** Specifications Table

Subject	Surgery; Perinatology, Pediatrics and Child Health
Specific subject area	Pediatric Surgery, Quality of Life, Duodenal Atresia
Type of data	Table
How data were acquired	Potential participants and their demographic, clinical and operative data was obtained from a prospectively maintained RedCAP database of patients with duodenal atresia (DA). Quality of Life outcomes were obtained via survey, using Pediatric Quality of Inventory (PedsQL™) surveys. Survey scores were converted to quality of life scores (out of 100) using Excel for Mac Version 16 (Redmond WA, Microsoft Corp).
Data format	Raw and analysed.
Parameters for data collection	Data points collected from the database were determined by the research team in consultation with local experts (consultant surgeons) in pediatric surgery. The PedsQL™ surveys was chosen as they are validated health related quality of life scoring systems with publically available control cohort data.
Description of data collection	Demographic and clinical data was collected from a prospectively maintained RedCAP database (DB#077) of all patients with duodenal atresia (DA) at our centre from July 2000 to current. Quality of life data was obtained from returned PedsQL™ 4.0 core score surveys and PedsQL™ gastrointestinal module surveys from children with duodenal atresia and their parents, whom were identified in the database.
Data source location	Department of Pediatric Surgery, The Royal Children's Hospital, Melbourne, Australia.
Data accessibility	Repository name: Mendeley Data https://doi.org/10.17632/837kr7tcy5.1 Direct URL to data: https://data.mendeley.com/datasets/837kr7tcy5/draft?a=89bf9a6b-9471-482c-9735-b295bf09def6
Related research article	Vinycomb et al., Quality of life outcomes in children born with duodenal atresia. Journal of Pediatric Surgery. *Accepted pending minor revisions* [1].

**Table:** 

**Value of the Data** • This prospectively collected data provides the demographic and clinical data, as well as quality of life outcomes, on patients with duodenal atresia. • Data would benefit researchers who are investigating the quality of life of pediatric gastrointestinal or congenital surgical conditions. • This raw data can be used as a foundation or comparison group for future quality of life studies that investigate congenital surgical conditions including duodenal atresia. This includes subsets of patients with other comorbidities, such as trisomy 21, that can affect quality of life. • Data can be used to contribute to the epidemiological studies of the anatomy and comorbidities of patients with duodenal atresia.

## 1 Data

This raw dataset (contained in the linked repository [2]) contains demographic information, as well as clinical, diagnostic and operative data for all participants (section 1) and non-participants (section 2). Also contained are the responses from each participating family (parent, and if applicable, child) for each question in the Pediatric Quality of Life Inventory (PedsQL™) 4.0 generic core score (section 3) and PedsQL™ gastrointestinal (GI) module (section 4).

Section 1 (participants, n = 38) and section 2 (non-participants, n = 72) contains demographic data including sex and the patients age at the time the study was commenced (May 30, 2018), converted from DOB to protect patient privacy. Clinical data includes gestation, birth weight and multiple gestation is also presented. Diagnostic information includes location of duodenal atresia (DA), description of pancreas, gastrointestinal rotation, associated syndromes, cardiac and chromosomal anomalies. Operative data includes type of repair, type of anastomosis and whether a Ladd's procedure was performed. For participants, converted scores for the four scales of the PedsQL™ core score are presented: physical, emotional, social and school functioning. Also included is the physical and psychosocial health summary scores.

## 2 Experimental design, materials, and methods

### 2.1 Database

This article presents data from a prospectively collected RedCAP database (DB#077) of patients with duodenal atresia (DA) at the Royal Children's Hospital, Melbourne, since July 2000. This database contains extensive detail of demographic, clinical (antenatal and postnatal), and operative information with over 500 specific data points. Participants were eligible for participation if they were ≥2 years at the time of the start of the study (30 May 2018) and had an operative diagnosis of duodenal atresia. Fifteen data points deemed relevant for this study were extracted from the database for all eligible participants (n = 120). All the extracted data points are reported in section 1 and section 2.

### 2.2 Quality of life outcome questionnaires

The PedsQL™ questionnaires are a collection of validated surveys that assess the quality of life in children. There are a variety of questionnaires that examine general quality of life, specific body systems (e.g. gastrointestinal or cardiac) or specific medical conditions (e.g. cerebral palsy or asthma). In this study we use the PedsQL™ 4.0 generic core score [3] and the PedsQL™ gastrointestinal symptoms module [4].

The core score questionnaires contains 23 items for young children (5–7 years), children (8–12 years) and teens (13–18 years). Parents and children have the same number of items covering the same four domains. For toddlers (2–4 years) there is only a parent questionnaire containing 21 items. The GI module also only contains a parent questionnaire for toddlers, and a parent and child questionnaire for the other age groups. Each GI questionnaire contains 74 items over 14 domains. All questionnaires cover the frequency of symptoms for the child over the last one month.

### 2.3 Survey collection

Contact information (address and telephone number) was retrieved from The Royal Children's Hospital (Melbourne, Australia) electronic medical record system. Patients who had not attended the hospital in the last five years were first sent a tracing letter as recommended by our local ethics committee. Participant's families that responded to the tracing letter, or that had been seen by our health service in the last five years were then invited to participate. These families were sent a PedsQL™ generic core scale and PedsQL™ GI module questionnaire appropriate for the patient's age. Surveys were returned by included postage-paid envelopes. If the survey was not returned in four weeks, two attempts were made to contact the family via phone. Families then given the option to return the physical survey or complete the survey over the phone.

### 2.4 Data analysis

Raw scores are provided as received by participants, with direct entry of the results from manually completed questionnaire forms. If more than one answer was marked for any given question, the result for that question was excluded from the study and marked as not answered (‘N/A’).

Raw scores were inversely scored then linear converted to a score from 0 to 100 using Excel for Mac Version 16 (Redmond, WA; Microsoft Corp) using the following Excel formula: =ABS(('raw score'×25)−100). The average score (Excel formula: =average('converted scores')) for each participant was then calculated as the overall score. The score for each scale in the core score (physical, emotional, social and school functioning scales) and GI module (see section 2) were calculated by averaging the answered questions for each domain. The psychosocial health summary score was calculated by averaging all the answered questions in the emotional, social and school functioning scales. The physical health summary score was the same as the physical functioning scale. As per the PedsQL™ scoring instructions [3], if more than 50% of items for any scale, or overall, were missing, the score was not calculated. Scores are presented on a linear scale of 0–100, with a higher score equating to a better quality of life.

Further data analysis is performed and presented in a separately published article by the same authors [1].

### 2.5 Ethics approval

Approval was obtained from this study from The Royal Children's Hospital Human Research Ethics Committee (HREC 38054A).
